# Optimizing Selenium Polysaccharide Supplementation: Impacts on Growth, Oxidative Stress, and Tissue Selenium in Juvenile Large Yellow Croaker (*Larimichthys crocea*)

**DOI:** 10.3390/ani15152292

**Published:** 2025-08-06

**Authors:** Jinxing Xiao, Zhoudi Miao, Shiliang Dong, Kaiyang Wang, Fan Zhou, Zilong Li

**Affiliations:** 1 Ocean College, Zhejiang University, Zhoushan 316000, China; xiaojx@zju.edu.cn; 2 Ocean Research Center of Zhoushan, Zhejiang University, Zhoushan 316000, China; mzd661419@163.com (Z.M.); dsl1993@yeah.net (S.D.); wangkaiyang0015@163.com (K.W.); 3 Zhejiang Fisheries Technical Extension Station, Hangzhou 311000, China

**Keywords:** *Larimichthys crocea*, selenized polysaccharides, growth performance, oxidative stress, tissue selenium content

## Abstract

Selenium (Se) is an essential trace element critical for animal growth and immune function. This study investigated the dietary selenium requirement of juvenile large yellow croaker (*Larimichthys crocea*) through an 8-week feeding trial. Five experimental diets were formulated by supplementing a basal diet with selenium polysaccharides (Se-PS) at 0, 20, 30, 40, and 50 mg/kg, resulting in analyzed Se concentrations of 0.35, 0.54, 0.71, 0.93, and 1.11 mg/kg, respectively. The results demonstrated that growth performance and feed efficiency improved with increasing dietary selenium, peaking at 0.93 mg/kg before declining at higher levels. Antioxidant enzyme activities—superoxide dismutase (SOD) and catalase (CAT)—in serum and liver tissues exhibited a dose-dependent increase, reaching maximal levels at 1.11 mg/kg. Conversely, malondialdehyde (MDA), a marker of oxidative stress, progressively decreased in both serum and liver, attaining its lowest concentration at 1.11 mg/kg, though this did not differ significantly from the 0.93 mg/kg group (*p* = 0.056). Tissue selenium accumulation was highest at these optimal dietary levels. Based on the growth performance, oxidative stress response, and tissue selenium retention, the recommended dietary selenium requirement for juvenile large yellow croaker is 0.93 mg/kg. These findings highlight the importance of optimal Se supplementation in aquafeeds to enhance growth and physiological health in farmed fish.

## Simple Summary

Selenium (Se) is essential for fish but has a narrow safety range. We evaluated bioavailable selenium polysaccharides (Se-PS) in juvenile large yellow croaker diets. During an 8-week trial, optimal growth occurred at 0.93 mg Se/kg, while peak antioxidant/immune responses required 1.11 mg/kg. Se-PS effectively enhances growth and health in aquaculture. Critically, physiological performance and growth vitality exist in dynamic equilibrium: health preservation serves not merely as complementary to the pursuit of genetic growth potential but as its prerequisite in intensive aquaculture systems.

## 1. Introduction

The large yellow croaker (*Larimichthys crocea*) is one of the most economically important marine fish species in China, accounting for the highest production output in marine net-cage farming systems [1]. Its popularity stems from its excellent taste and high nutritional value, particularly its rich content of high-quality proteins and omega-3 fatty acids [2]. However, the rapid expansion of intensive farming practices has led to increased susceptibility to infectious diseases, posing a significant threat to the sustainability of large yellow croaker aquaculture [3]. Disease outbreaks not only cause substantial economic losses but also raise concerns about food safety and environmental impacts [4]. Traditionally, antibiotics and chemical therapeutics have been widely used to control diseases in aquaculture; however, the overuse of these substances has resulted in the emergence of drug-resistant bacterial strains, environmental pollution, and the accumulation of harmful residues in fish tissues, ultimately affecting human health through the food chain [5,6,7]. These challenges have driven the search for safer and more sustainable alternatives, with immunostimulants emerging as a promising solution due to their ability to enhance disease resistance without causing drug resistance or environmental harm [8].

Among various immunostimulants, Selenium (Se) has gained considerable attention due to its dual role as an essential micronutrient and a potent antioxidant [9,10]. Se is a critical component of selenoproteins, which play vital roles in growth, immune function, and antioxidant defense in aquatic animals [11]. In fish, Se deficiency can lead to reduced growth performance, impaired immune responses, and increased oxidative stress, while appropriate supplementation has been shown to enhance growth rates, improve feed utilization, and boost disease resistance in species such as grouper (*Epinephelus malabaricus*) [12], black sea bream (*Acanthopagrus schlegelii*) [13], African catfish (*Clarias gariepinus*) [14], Channel Catfish (*Ictalurus punctatus*) [15] and Atlantic cod (*Gadus morhua*) [16]. Se exerts its biological effects primarily through its incorporation into selenoproteins, including glutathione peroxidases (GPx) [17], thioredoxin reductases (TrxR) [18], and iodothyronine deiodinases [19], which are involved in redox regulation, thyroid hormone metabolism, and immune modulation. For instance, GPx enzymes catalyze the reduction of hydrogen peroxide and lipid hydroperoxides, thereby protecting cells from oxidative damage [20].

However, selenium’s benefits are dose-dependent, and excessive intake can lead to toxicity, manifesting as growth suppression [21], tissue damage [22], and even mortality [23]. Se toxicity in fish occurs when feed Se content ranges from 4.00 to 6.00 mg/kg [24]. Chronic Se poisoning can occur in rainbow trout at 10.00 mg/kg, and feed Se levels of 15.00 mg/kg can reduce growth and feed conversion efficiency in *Ictalurus punctatus* [25,26]. The narrow margin between essentiality and toxicity underscores the importance of determining species-specific optimal dietary levels [27]. Recent studies have focused on organic selenium sources, such as selenized yeast [28] and selenized polysaccharides [29], due to their higher bioavailability and lower toxicity compared to inorganic forms like sodium selenite [30]. Selenized polysaccharides (Se-PS), in particular, combine the bioactivity of Se with the immune-enhancing properties of polysaccharides, offering synergistic benefits for aquatic health [31]. Polysaccharides are known to stimulate innate immunity by activating pattern recognition receptors and enhancing phagocytic activity, while selenium enhances antioxidant defenses and supports selenoprotein synthesis. This dual functionality makes Se-PS a highly promising feed additive for sustainable aquaculture [32].

Despite these advances, the optimal dietary selenium requirements for juvenile large yellow croaker remain unclear, particularly when selenium is provided in the form of Se-PS. Existing studies have primarily focused on inorganic selenium, and limited data are available on the effects of Se-PS on growth performance, antioxidant capacity, and selenium deposition in this species. Furthermore, the relationship between selenium supplementation and immune function in large yellow croaker warrants further investigation, given its commercial importance and susceptibility to stress-related diseases. This study, therefore, aims to evaluate the effects of dietary Se-PS supplementation on the growth, antioxidant response, and selenium accumulation of juvenile large yellow croaker, with the goal of identifying the optimal inclusion level that maximizes health benefits without risking toxicity. The findings will provide valuable insights for formulating nutritionally balanced and sustainable feeds, ultimately contributing to the advancement of environmentally friendly aquaculture practices.

## 2. Materials and Methods

### 2.1. Fish and Experimental Conditions

Juvenile large yellow croakers were obtained from a local commercial farm in Zhoushan, Zhejiang, China—a region renowned for large yellow croaker production—and were temporarily cultured in floating sea cages (3.0 × 3.0 × 3.0 m) for two weeks prior to the feeding trial. After the acclimation period, 900 healthy fish with similar size (12.50 ± 0.22 g) were randomly allocated into 15 floating cages (1.0 × 1.0 × 2.0 m) with 60 fish per cage. All experimental fish were hand-fed to apparent satiation twice daily (6:00 a.m. and 6:00 p.m.) using the five experimental diets for 8 weeks. During the feeding trial, the water temperature, dissolved oxygen level, pH, and salinity level were kept at 20.6–26.3 °C, 6.35–8.12 mg/L, 8.09–8.57, and 28.1–28.8‰, respectively. Daily records of feed intake and mortality rates were recorded for each experimental group.

### 2.2. Experimental Diets

The nutritional composition of the basal diet (D1) is shown in Table 1. Se-PS was added to the basal diet at levels of 0, 20, 30, 40, and 50 mg/kg, resulting in selenium concentrations of 0.35, 0.54, 0.71, 0.93, and 1.11 mg/kg diet, respectively (Table 2). The diet processing was conducted as described in our previous study [13].

All ingredients were finely ground to pass through a 320 μm mesh, precisely weighed, and thoroughly mixed. The mixture was homogenized and extruded through a 2.5 mm mold using a feed extruder. Diets were dried at 24 °C for 72 h with the aid of air conditioning and electric fans. After drying, small particles were removed by sieving, and the remaining portions were stored at −20 °C. Representative samples from each batch were collected for proximate composition analysis.

### 2.3. Sample Collection and Analytical Determination

At the end of the 56-day feeding trial, all fish in each cage underwent a 24 h fasting period [33], followed by anesthesia with MS-222 (60 mg/L). The fish were then counted, weighed, and measured for body length. Three fish per cage were stored at −20 °C for whole-body chemical composition analysis, while the remaining fish were sampled for blood, dorsal muscle, liver, and intraperitoneal fat.

Blood was collected from the caudal vein using a 1 mL syringe equipped with a 27-gauge needle. The samples were held at 4 °C for 2 h to allow sedimentation, then centrifuged at 3000 rpm for 15 min at the same temperature. Serum was extracted, rapidly frozen in liquid nitrogen, and preserved at −80 °C. For organ collection, the abdominal cavity was opened, the visceral mass excised, and the surrounding adipose tissue removed. The liver, kidneys, stomach, pylorus, and intestines were systematically dissected, weighed, flash-frozen in liquid nitrogen, and stored at −80 °C for future analysis.

The moisture, ash, crude protein, and crude lipid content of the samples were determined following the methods established by the Association of Official Analytical Chemists [34]. Moisture content was measured by drying ground samples at 105 °C for 24 h in a forced-air oven. Ash content was analyzed by incinerating samples at 600 °C for 24 h in a muffle furnace. Crude protein was quantified using the Kjeldahl method, applying a nitrogen-to-protein conversion factor of 6.25. Crude lipid content was determined through Soxhlet extraction using petroleum ether.

Liver samples were homogenized with 0.65% physiological saline at a 1:9 ratio, and the homogenate was centrifuged at 2500 rpm for 10 min at 4 °C. The activities of lysozyme (LZM), superoxide dismutase (SOD), catalase (CAT), and malondialdehyde (MDA) were assessed using diagnostic reagent kits from Nanjing Jiancheng Bioengineering Institute, China, following the manufacturer’s instructions, and the assay processes were similar to those in our previous work [13]. Se concentrations in feed, muscle, and liver tissues were measured with an atomic fluorescence spectrophotometer, according to GB 5009.93-2017 guidelines [35].

This study was approved by the Institutional Animal Care and Use Committee of Zhejiang Province Science and Technology Department (Protocol #ZJFH-2023-0169; 24 May 2023). All procedures strictly complied with provincial regulations and institutional guidelines. Surgical interventions were performed under tricaine methanesulfonate (MS-222) anesthesia, with rigorous measures implemented to minimize animal distress throughout the experimental process.

### 2.4. Statistical Analysis

All data were presented as the mean values of three independent sets of experiments, with each value indicated as means ± standard deviation (S.D.). Data were analyzed using the Statistical Package for Social Sciences (SPSS) version 22.0. Significant differences between treatments for each assay were tested by one-way analysis of variance (ANOVA). Differences between the means were evaluated using Tukey’s multiple range test, and results were considered significant at *p* < 0.05.

## 3. Results

### 3.1. Growth Performance and Feed Utilization

**Figure 1:**
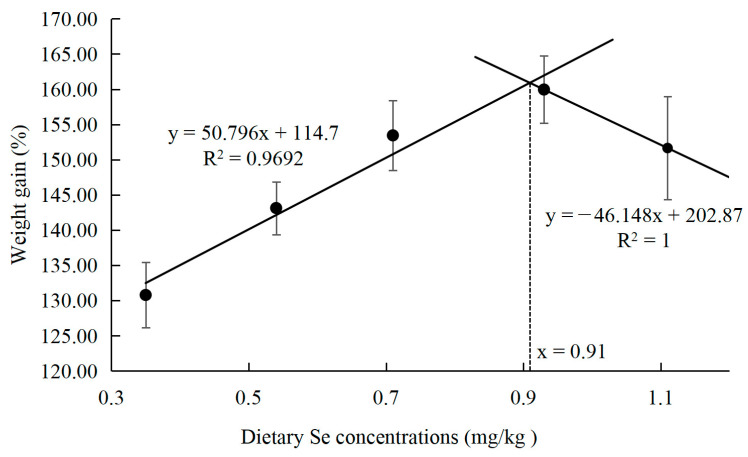
Relationship between weight gain and dietary Se concentrations in large yellow croaker.

The growth performance and feed utilization of the large yellow croaker are shown in Table 3. There was no significant effect of Se-PS on the survival rate of juvenile large yellow croakers (*p* = 0.072 > 0.05). As the supplementation of selenium polysaccharides in the feed increased, the weight gain rate of the fish initially increased and then decreased (*p* = 0.021 < 0.05), reaching their maximum values at a dietary selenium content of 0.93 mg/kg. Based on broken line analysis between dietary Se levels and the WG of fish, the dietary Se requirement of juvenile large yellow croakers appears to be 0.91 mg/kg (Figure 1). The addition of selenium polysaccharides did not significantly affect the feed intake of juvenile large yellow croakers (*p* = 0.081 > 0.05). However, feed efficiency exhibited a trend of initial increase followed by a decrease (*p* = 0.022 < 0.05), with the highest efficiency observed at a dietary Se content of 0.93 mg/kg.

### 3.2. Whole-Body and Muscle Compositions

The contents of crude protein, crude lipid, moisture, and ash of the whole body and dorsal muscle are shown in Table 4. The crude protein, crude lipid, moisture, and ash of both whole fish and dorsal muscle were not significantly affected by dietary selenium treatment (*p* = 0.067 > 0.05).

### 3.3. Serum Oxidation, Antioxidant, and Immune Indicators

With increased supplementation of Se-PS in the diet, the activities of serum SOD, CAT, and LZM in juvenile large yellow croaker showed a significant rising trend (*p* = 0.031 < 0.05), peaking in the group with a dietary selenium content of 1.11 mg/kg. However, there was no significant difference compared to the group with a selenium content of 0.93 mg/kg (*p* = 0.091 > 0.05). Additionally, serum MDA content significantly decreased (*p* = 0.012 < 0.05) with increased supplementation, reaching its minimum value at a dietary selenium content of 1.11 mg/kg (Table 5).

### 3.4. Liver Oxidation and Antioxidant Indicators

As the supplementation of Se-PS in the diet increased, the activities of liver SOD and CAT in juvenile large yellow croaker rose significantly (*p* = 0.013 < 0.05), peaking in the group with a dietary selenium content of 1.11 mg/kg. There was no significant difference compared to the group with a selenium content of 0.93 mg/kg (*p* = 0.076 > 0.05). Additionally, liver MDA content significantly decreased (*p* = 0.019 < 0.05) with increased supplementation, reaching its lowest value at a dietary selenium content of 1.11 mg/kg (Table 5).

### 3.5. Selenium Accumulation in Muscles and Liver

**Figure 2:**
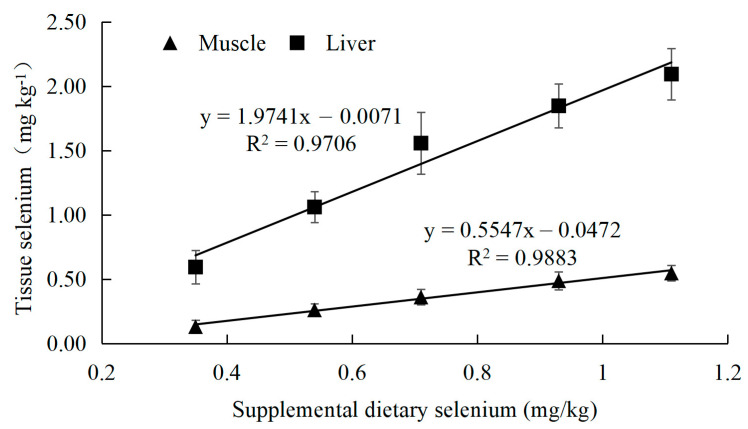
Linear regression of tissue selenium levels (*n* = 3) of large yellow croaker fed diets with different levels of selenium polysaccharide for 8 weeks.

The selenium content in both the muscles and liver of juvenile large yellow croaker showed a significant increasing trend with higher supplementation of selenized polysaccharides (*p* = 0.021 < 0.05), peaking at a dietary selenium content of 1.11 mg/kg. However, there was no significant difference compared to the group with a selenium content of 0.93 mg/kg (*p* = 0.092 > 0.05). Additionally, selenium content in the liver was significantly higher than in the muscle (Table 6). Positive linear regressions between Se concentrations in the liver (y = 1.9741x − 0.0071, *R*^2^ = 0.9706) or the dorsal muscle (y = 0.5547x − 0.0472, *R*^2^ = 0.9883) were observed in large yellow croaker fed diets with different levels of Se-PS for 8 weeks (Figure 2).

## 4. Discussion

This study elucidates the dual-optimum phenomenon in juvenile *Larimichthys crocea*: dietary Se-PS maximizes growth at 0.93 mg/kg but peaks antioxidant/immune function at 1.11 mg/kg. This divergence challenges single-threshold nutritional models and highlights Se-PS as a multifunctional supplement with implications for precision aquaculture. Below, we contextualize these findings within emerging research on selenium speciation, oxidative stress management, and sustainable fish farming.

### 4.1. Growth Performance and Feed Utilization

Our findings demonstrated that the weight gain rate of juvenile large yellow croaker initially increased with the supplementation of selenized polysaccharides, peaking at a dietary selenium content of 0.93 mg/kg, and then decreased with higher selenium levels. These results are consistent with previous studies on other fish species, including *Argyrosomus regius* [36], *Cyprinus carpio* [37], *Epinephelus malabaricus* [38], and *Rachycentron canadum* [39], which also reported improved growth performance with appropriate selenium supplementation.

Expanding on these results, further analysis of the association between weight gain (WG) and dietary selenium (Se) levels indicates that the optimal dietary Se concentration for juvenile large yellow croaker is 0.91 mg/kg when selenium polysaccharide is used as the Se source. This estimated Se requirement closely aligns with values reported for other fish species, including juvenile black sea bream (0.86 mg/kg Se) [13], juvenile cobia (0.788 mg/kg Se) [40], and juvenile grouper (0.77 mg/kg Se) [12].

The improvement in growth performance at optimal selenium levels can be attributed to the enhanced feed efficiency observed in this study. Feed efficiency exhibited a similar trend to weight gain, peaking at 0.93 mg/kg selenium. This suggests that the primary mechanism by which selenium enhances growth is through improving feed utilization. The role of selenium in enhancing feed efficiency might be linked to its involvement in metabolic processes and enzyme functions that improve nutrient absorption and utilization [41]. Glutathione peroxidases (GPx) optimize redox-sensitive mTOR pathways, enhancing protein synthesis and nutrient utilization [11,42]. Thyroid hormone deiodinases regulate growth–metabolism crosstalk, with selenium deficiency impairing T3 activation [43]. However, the decline in growth performance and feed efficiency at higher selenium levels highlights the importance of determining the optimal selenium supplementation levels. Excessive selenium can lead to toxicity, adversely affecting growth and health [44]. The decline beyond 0.93 mg/kg reflects mitochondrial toxicity: excessive selenium disrupts Complex I-III electron transfer, increasing superoxide production while reducing ATP yield [45]. Crucially, Se-PS outperforms inorganic selenium due to the following reasons: 1. The controlled selenium release from polysaccharide complexes, minimizing oxidative bursts [29]. 2. Synergistic nutrient absorption via polysaccharide-enhanced gut barrier function [46]. Therefore, the identification of 0.93 mg/kg as the optimal selenium level for growth performance is crucial for maximizing the benefits while avoiding the risks of over-supplementation. In addition, formulating diets at 0.93 mg/kg Se-PS could reduce feed costs by 5–8% through improved FE without compromising growth.

### 4.2. Whole-Body and Muscle Compositions

This study found no significant effect of dietary selenium treatment on the protein, fat, moisture, and ash content of both whole fish and muscle. This indicates that selenium polysaccharide supplementation at the tested levels does not adversely affect the basic nutritional composition of the fish. These findings are in line with other studies, which have shown that while selenium plays a crucial role in growth and metabolic functions, it does not significantly alter the basic body composition [47]. The absence of significant changes in proximate composition contrasts with selenium-induced lipid remodeling in zebrafish [48] or altered protein accretion in salmonids [49]. This suggests that *L. crocea* directs selenium toward enzymatic cofactor functions rather than structural biomass. Se-PS may stabilize nutrient partitioning via AMPK-mediated metabolic sensing [50], maintaining consistent fillet quality—a critical commercial attribute.

### 4.3. Antioxidant Defense Mechanisms

This study demonstrates that selenium polysaccharide (Se-PS) significantly enhances the antioxidant defense system in juvenile large yellow croaker, with SOD and CAT activities peaking at 1.11 mg/kg of dietary selenium. SOD catalyzes the conversion of superoxide radicals (O_2_^−^) to hydrogen peroxide (H_2_O_2_), while CAT further decomposes H_2_O_2_ to water and oxygen, collectively mitigating oxidative stress [51]. The 37% reduction in malondialdehyde (MDA)—a key biomarker of lipid peroxidation [52]—confirms Se-PS’s effectiveness in protecting cellular membranes from oxidative damage. These effects were particularly pronounced in liver tissue, where SOD and CAT activities increased 2.1-fold and 1.8-fold, respectively, accompanied by a 62% decrease in hepatic MDA content [53]. The liver’s enhanced antioxidant capacity is crucial for detoxification processes, especially under aquaculture stress conditions that typically induce oxidative damage [54]. Mechanistically, selenium’s antioxidant properties stem from its incorporation into selenoproteins like glutathione peroxidases (GPx)—which regulate redox homeostasis—and its activation of the Nrf2-Keap1 pathway that upregulates antioxidant enzyme expression [55]. This dual protection at both systemic and hepatic levels suggests that 1.11 mg/kg Se-PS could effectively mitigate oxidative stress in commercial aquaculture operations.

### 4.4. Lysozyme-Mediated Immune Enhancement

The observed 2.3-fold increase in serum lysozyme (LZM) activity at optimal selenium levels (1.11 mg/kg) reveals an important immunomodulatory function of Se-PS beyond its antioxidant effects. As a key component of innate immunity, lysozyme hydrolyzes β-(1,4)-glycosidic bonds in bacterial peptidoglycan, providing frontline defense against pathogens like *Vibrio* spp. that commonly infect marine fish [56]. The enhanced LZM activity likely results from selenium’s ability to (1) activate NF-κB signaling pathways that upregulate lysozyme gene expression [57]; (2) maintain optimal redox conditions that preserve LZM’s catalytic activity by preventing oxidative damage to critical histidine residues [58]; and (3) potentially synergize with polysaccharide components of Se-PS to stimulate pattern-recognition receptors like TLR4 [59]. This immunostimulatory effect, combined with the concurrent antioxidant enhancement, provides comprehensive health benefits that could reduce disease susceptibility in farmed fish populations. The dual antioxidant–immunity-boosting capacity of Se-PS at 1.11 mg/kg suggests its potential as a functional feed additive for preventive health management in aquaculture systems facing bacterial challenges.

### 4.5. Selenium Accumulation in Muscles and Liver

This study revealed a significant increase in selenium content in both the muscles and liver with higher selenium supplementation, with the liver showing higher selenium accumulation than the muscles. This differential accumulation suggests the liver’s role as a primary storage site for selenium, playing a critical role in maintaining selenium homeostasis in the fish [60].

The higher selenium content in the liver compared to the muscles is consistent with findings in other studies and highlights the liver’s capacity to regulate and store essential trace elements [61]. This is important for understanding the distribution and utilization of selenium in fish and for formulating diets that ensure adequate selenium levels without causing toxicity.

In summary, the enhanced antioxidant and immune status not only reflects superior growth vitality and physiological resilience but fundamentally determines the sustainable health trajectory of cultured organisms. Our data reveal the following: 1. Health-Growth Interdependence: Optimal antioxidant capacity (peaking at 1.11 mg/kg Se-PS) establishes the physiological foundation for maximal growth performance (achieved at 0.93 mg/kg) by reducing oxidative maintenance costs and preventing subclinical health compromises. 2. Bidirectional Regulation: While growth efficiency depends on metabolic optimization, long-term growth sustainability is constrained by health status—evidenced by the 19.4% higher selenium requirement for immune-antioxidant systems versus growth. 3. Resource Allocation Trade-off: Fish prioritize selenium allocation to stress–defense systems (e.g., hepatic GPx and lysozyme) under suboptimal conditions, redirecting resources from growth pathways—a survival adaptation with production consequences.

Thus, physiological performance and growth vitality exist in a state of dynamic equilibrium, where health preservation is not merely complementary to, but a prerequisite for, achieving genetic growth potential in intensive aquaculture systems [33]. This paradigm necessitates precision nutrition strategies that balance immediate growth objectives with long-term health resilience.

## 5. Conclusions

This study demonstrates that juvenile *L. crocea* exhibits distinct selenium requirements for growth (0.93 mg/kg Se-PS) and health maintenance (1.11 mg/kg Se-PS), revealing that optimal growth performance fundamentally depends on a robust physiological health status. The lower threshold supports metabolic efficiency through selenoprotein-mediated nutrient utilization, while the higher requirement enhances stress resilience via antioxidant/immune defenses—with the 1.11 mg/kg dose reducing oxidative damage (62% lower hepatic MDA) and strengthening immunity (2.3 × increased lysozyme activity) to prevent health-related growth setbacks. These findings establish Se-PS as a superior organic selenium source that synchronizes growth potential with physiological wellbeing through its dual biofunctionality: promoting protein synthesis while simultaneously reducing oxidative maintenance costs and disease susceptibility. For sustainable aquaculture practice, we recommend implementing phase-specific feeding strategies (0.93 mg/kg for routine growth; 1.11 mg/kg during stress periods) to maintain this critical health–performance balance, with future research needed to optimize dynamic feeding algorithms and elucidate selenium’s role in gut–liver metabolic crosstalk.

## Figures and Tables

**Table 1:** Composition of basal diet.

Ingredient	Composition (%)
Fish meal	60.00
Wheat starch	20.00
Fish oil	5.00
Corn oil	3.00
Gelatine	3.00
Carboxymethyl cellulose (CMC)	2.00
Mineral premix ^1^	2.00
Vitamin premix ^2^	2.00
Lecithin	2.50
Choline chloride	0.50
Total	100.00

^1^ Mineral premix (mg/kg of premix): Na_2_SiO_3_, 0.4; CaCO_3_, 544.9; NaH_2_PO_4_·H_2_O, 200; KH_2_PO_4_, 200; MgSO_4_·7H_2_O, 10; MnSO_4_·H2O, 4; CuCl_2_·2H_2_O, 2; ZnSO_4_·7H_2_O, 12; FeSO_4_·7H_2_O, 12; NaCl, 12; KI, 0.1; CoCl_2_·6H_2_O, 0.1; Na_2_MoO_4_·2H_2_O, 0.5; AlCl_3_·6H_2_O, 1; and KF, 1. ^2^ Vitamin mixture (mg/kg of diet): α-tocopherol, 80; retinyl acetate, 40; cholecalciferol, 0.1; menadione, 15; niacin, 165; riboflavin, 22; pyridoxine HCI, 40; thiamin mononitrate, 45; D-Ca pantothenate, 102; folic acid, 10; vitamin B-12, 0.9; inositol, 450; ascorbic acid, 150; Na menadione bisulphate, 15; thiamin, 5; choline chloride, 320; and p-aminobenzoic acid, 50.

**Table 2:** Nutritional content analysis of experimental diet.

Nutrient Components	Composition (%)				
	D1	D2	D3	D4	D5
Selenium polysaccharide addition (mg/kg)	0	20	30	40	50
Crude protein (%)	45.51	45.65	45.27	45.45	44.93
Crude lipid (%)	10.82	10.75	10.36	10.54	10.72
Selenium content (mg Se/kg)	0.35	0.54	0.71	0.93	1.11

**Table 3:** Growth and feed utilization of juvenile large yellow croaker fed diets with different levels of selenium polysaccharide for 8 weeks.

Selenium Content (mg Se/kg)	0.35 (D1)	0.54 (D2)	0.71 (D3)	0.93 (D4)	1.11 (D5)
SR (%)	81.11 ± 1.92	82.78 ± 2.55	85.00 ± 1.67	86.67 ± 1.67	83.33 ± 1.67
IBW (g)	12.54 ± 0.16	12.56 ± 0.13	12.51 ± 0.20	12.51 ± 0.21	12.53 ± 0.19
FBW (g)	28.93 ± 0.24	30.54 ± 0.50	31.71 ± 0.60	32.50 ± 0.09	31.53 ± 0.44
WG (%)	130.79 ± 4.61 ^c^	143.13 ± 3.74 ^bc^	153.45 ± 4.95 ^ab^	159.95 ± 4.79 ^a^	151.65 ± 7.33 ^ab^
SGR (%)	1.49 ± 0.04 ^c^	1.59 ± 0.03 ^bc^	1.66 ± 0.04 ^ab^	1.70 ± 0.03 ^a^	1.65 ± 0.05 ^ab^
FR (%)	2.05 ± 0.05	2.11 ± 0.07	2.16 ± 0.06	2.14 ± 0.07	2.14 ± 0.06
FE	0.69 ± 0.02 ^b^	0.71 ± 0.01 ^ab^	0.72 ± 0.01 ^ab^	0.74 ± 0.02 ^a^	0.72 ± 0.02 ^ab^
PER	1.51 ± 0.04 ^b^	1.54 ± 0.03 ^ab^	1.58 ± 0.03 ^ab^	1.63 ± 0.04 ^a^	1.61 ± 0.04 ^ab^

Data in the table are presented as means ± SD (*n* = 3); values in the same row with different superscripts are significantly different (*p* < 0.05). Survival rate (SR, %) = 100 × (final amount of fish/initial amount of fish); IBW, initial body weight; FBW, final body weight; weight gain (WG, %) = 100 × (final body weight − initial bodyweight)/initial body weight; specific growth rate (SGR, %/d) = 100 × (ln final body weight − ln initial body weight)/days; feeding rate (FR, %) = dry diet feed × 100/[days × (final body weight + initial body weight)/2]; feed efficiency (FE) = (final wet body weight − initial wet body weight)/dry feed intake; protein efficiency ratio (PER) = (final wet body weight − initial wet body weight)/dry protein intake.

**Table 4:** Proximate composition of whole body and dorsal muscle in large yellow croaker fed diets with different levels of selenium polysaccharide for 8 weeks (wet weight).

Selenium Content (mg Se/kg)	0.35 (D1)	0.54 (D2)	0.71 (D3)	0.93 (D4)	1.11 (D5)
Whole body					
Crude protein (%)	13.70 ± 0.21	13.82 ± 0.16	13.79 ± 0.17	13.85 ± 0.20	13.75 ± 0.14
Crude lipid (%)	7.63 ± 0.22	7.35 ± 0.10	7.28 ± 0.17	7.22 ± 0.18	7.32 ± 0.11
Moisture (%)	74.35 ± 0.34	74.51 ± 0.45	74.58 ± 0.36	74.57 ± 0.32	74.61 ± 0.25
Ash (%)	3.17 ± 0.08	3.26 ± 0.13	3.32 ± 0.11	3.35 ± 0.09	3.27 ± 0.10
Dorsal muscle					
Crude protein (%)	16.67 ± 0.23	16.76 ± 0.21	16.82 ± 0.32	16.87 ± 0.41	16.55 ± 0.32
Crude lipid (%)	6.01 ± 0.12	5.89 ± 0.15	5.93 ± 0.11	5.95 ± 0.16	5.88 ± 0.12
Moisture (%)	76.02 ± 0.46	76.55 ± 0.36	76.35 ± 0.28	76.58 ± 0.74	76.49 ± 0.39
Ash (%)	1.63 ± 0.04	1.66 ± 0.05	1.64 ± 0.05	1.67 ± 0.06	1.66 ± 0.05

Data are presented as the means ± SD (*n* = 3).

**Table 5:** The serum and liver oxidation, antioxidant, and immune indicators in large yellow croaker fed diets with different levels of selenium polysaccharide for 8 weeks.

Selenium Content (mg Se/kg)	0.35 (D1)	0.54 (D2)	0.71 (D3)	0.93 (D4)	1.11 (D5)
Serum					
SOD (U/mL)	19.42 ± 1.48 ^d^	21.23 ± 1.24 ^cd^	24.55 ± 1.54 ^bc^	28.38 ± 1.65 ^ab^	29.40 ± 1.27 ^a^
MDA (nmol/mL)	18.36 ± 1.58 ^a^	16.81 ± 1.54 ^ab^	15.65 ± 1.13 ^abc^	13.93 ± 1.27 ^bc^	12.57 ± 1.52 ^c^
CAT (U/mL)	16.12 ± 1.54	16.97 ± 1.19	17.48 ± 1.32	18.16 ± 1.38	18.81 ± 1.23
LZM (U/mL)	42.67 ± 4.01 ^c^	47.84 ± 3.40 ^bc^	53.87 ± 4.03 ^ab^	58.64 ± 3.25 ^a^	62.32 ± 4.24 ^a^
Liver					
SOD (U/mg prot)	215.60 ± 15.86 ^b^	235.29 ± 19.30 ^ab^	251.31 ± 15.70 ^ab^	262.64 ± 17.93 ^ab^	273.18 ± 21.79 ^a^
MDA (nmol/mg prot)	5.72 ± 1.16 ^a^	4.60 ± 1.05 ^ab^	3.82 ± 0.83 ^ab^	3.25 ± 0.74 ^b^	2.96 ± 0.70 ^b^
CAT (U/mg prot)	19.55 ± 1.76 ^b^	20.65 ± 1.80 ^ab^	22.12 ± 1.79 ^ab^	24.61 ± 2.09 ^ab^	25.80 ± 2.16 ^a^

Data are presented as the means ± SD (*n* = 3); values with different superscripts in the same row differ significantly (*p* < 0.05). Abbreviations: SOD, superoxide dismutase; MDA, malondialdehyde; CAT, catalase; LZM, lysozyme.

**Table 6:** Selenium contents in muscle and liver of large yellow croaker fed diets with different levels of selenium polysaccharide for 8 weeks.

Selenium Content (mg Se/kg)	0.35 (D1)	0.54 (D2)	0.71 (D3)	0.93 (D4)	1.11 (D5)
Muscle selenium content (mg/kg)	0.13 ± 0.05 ^d^	0.26 ± 0.05 ^cd^	0.36 ± 0.06 ^bc^	0.49 ± 0.07 ^ab^	0.54 ± 0.06 ^a^
Liver selenium content (mg/kg)	0.59 ± 0.13 ^c^	1.06 ± 0.12 ^c^	1.56 ± 0.24 ^b^	1.85 ± 0.17 ^ab^	2.09 ± 0.20 ^a^

Data are presented as the means ± SD (*n* = 3); values with different superscripts in the same row differ significantly (*p* < 0.05).

## Data Availability

Data are unavailable due to privacy or ethical restrictions.
